# Multivalent Presentation of Ice Recrystallization Inhibiting Polymers on Nanoparticles Retains Activity

**DOI:** 10.1021/acs.langmuir.8b01952

**Published:** 2018-08-22

**Authors:** Christopher Stubbs, Laura E. Wilkins, Alice E. R Fayter, Marc Walker, Matthew I. Gibson

**Affiliations:** † Department of Chemistry, University of Warwick, Coventry CV4 7AL, United Kingdom; ‡ Department of Physics, University of Warwick, Coventry CV4 7AL, United Kingdom; § Warwick Medical School, University of Warwick, Coventry CV4 7AL, United Kingdom

## Abstract

Poly(vinyl alcohol) (PVA) has emerged as the most potent mimic of antifreeze (glyco)proteins ice recrystallization inhibition (IRI) activity, despite its lack of structural similarities and flexible, rather than rigid, backbone. The precise spacing of hydroxyl groups is hypothesized to enable PVA to recognize the prism planes of ice but not the basal plane, due to hydroxyl pattern matching of the ice surface giving rise to the macroscopic activity. Here, well-defined PVA derived from reversible addition–fragmentation chain-transfer (RAFT) polymerization is immobilized onto gold nanoparticles to enable the impact of nanoscale assembly and confinement on the observed IRI activity. Unlike previous reports using star-branched or bottle-brush PVAs, the nanoparticle–PVA retains all IRI activity compared to polymers in solution. Evidence is presented to show that this is due to the low grafting densities on the particle surface meaning the chains are free to explore the ice faces, rather than being constrained as in star-branched polymers. These results demonstrate a route to develop more functional IRI’s and inclusion of metallic particle cores for imaging and associated applications in cryobiology.

## Introduction

Nature has evolved a diverse range of biological macro-molecules which can modulate the formation and growth of ice to enable extremophiles to survive at subzero temperatures.^1^,^2^ The range of macromolecules known to have this function includes antifreeze proteins (AFPs),^1^ antifreeze glycoproteins (AFGPs),^3^ lipopolysaccharides,^4^ and polysaccharides.^5^ The exact mechanism of action, and the extent of actual ice binding, is not clear^6^ with different proteins targeting different crystallographic faces of ice, either through hydrophobic interactions in AFGPs^7^ or by anchored clathrates in some AFPs.^8^ Despite the challenges of studying ice/water interfacial processes and the current knowledge gaps, it has emerged that synthetic polymers can be designed to reproduce AF(G)P (antifreeze (glyco)protein) properties, even without any structural similarities to native antifreeze proteins.^9^–^11^ In particular, ice recrystallization inhibition (IRI; slowing ice growth rates) activity can be selectively retained, over thermal hysteresis/dynamic ice shaping, implying multiple mechanisms of action.^12^ As examples, safranin-O self-assembles into IRI active fibers,^13^ and short glycopeptides have been investigated by Ben et al. as AFGP mimics;^14^–^16^ poly(ampholytes) have weak IRI,^17^,^18^ and poly(vinyl alcohol) (PVA) has remarkably high IRI activity comparable to the shortest AFGPs.^19^–^22^ IRI activity is particularly desirable as ice crystal growth during thawing is a major cause of cell death during the cryopreservation of donor cells/tissues. Carpenter et al. first demonstrated that modulation of ice growth during thawing could improve the cryopreservation of red blood cells^23^ but that excessive dynamic ice shaping (a secondary property of AF(G)Ps) limited the concentration which could be applied due to formation of spicular ice morphologies which damaged cell membranes.^24^,^25^ Gibson and co-workers have demonstrated that polymeric IRIs can be used to significantly improve the cryopreservation of blood cells,^22^,^26^ nucleated cells,^27^ and even cell monolayers.^28^

Despite this diverse range of materials shown to have IRI activity, there is an outstanding question of how the different natural and synthetic antifreeze macromolecules interact with ice. We do not know the roles of bound and unbound components,^6^,^29^ if all ice-binding proteins have the same mechanism, or even if multiple molecular level mechanisms can give rise to similar macroscopic effects. For example, AFPs are rigid and have identifiable ice-binding faces (or water-structuring faces) which lead to ice binding. AFGPs, however, are more flexible^30^ and may bind via their peptide backbone,^7^ with the hydrophilic carbohydrate hydroxyls groups pointing away. This is in contrast to PVA, which is hypothesized to hydrogen bond via its hydroxyls to the ice surface.^31^,^32^ To further complicate the picture, synthetic materials with significant amphipathic character (opposing hydrophilic/hydrophobic face)^28^,^33^,^34^ but no obvious ice-binding sites, such as graphene oxide^35^ and metallohelices,^36^ also inhibit ice growth, supporting a mechanism where they sit at the quasiliquid layer and slow the rate of water transfer.^37^

An advantage of synthetic over natural polymers is the ability to tune their architecture^38^,^39^ (e.g., stars, brushes, cycles) to modulate function. Gibson et al. used reversible addition–fragmentation transfer (RAFT) polymerization and column chromatography to obtain ultra-low-dispersity PVA, showing that IRI activity activates above 10 repeat units in length^20^,^40^ and that block copolymerization,^41^ but not statistical copolymerization, is a tolerated modification supporting the hydroxyl hydrogen-bonding to ice model of Koop.^31^ Voets et al. found that bottle-brush PVA had no molecular-weight enhancement effects suggesting confinement of the PVA chains limits prism-face binding,^42^ and Congdon et al. showed that 3-arm star polymers have equal activity to 2 arms, despite having higher molecular weight.^43^ Interestingly, Type I AFPs are known to oligomerize to produce hyperactive variants, which can be considered to be the protein equivalent of varying polymer architecture to enhance activity.^44^ There is clearly a complex relationship between macromolecular architecture and IRI, and the design of more complex materials with multifunctional properties, such as imaging modalities, requires more detailed studies both to help translation but also to understand the mechanism of action.

Considering the above, in this work we designed and synthesized metal-core nanoparticles bearing multiple copies of poly(vinyl alcohol) on their surface, using RAFT polymerization to enable control over molecular weight and to install a thiol-anchoring group. We identify polymers which lead to colloidally stable gold particle dispersions to enable the ice recrystallization activity and ice shaping activity of the hybrids to be quantified and compared to free polymers in solution.

## Results and Discussion

**Figure 1:**
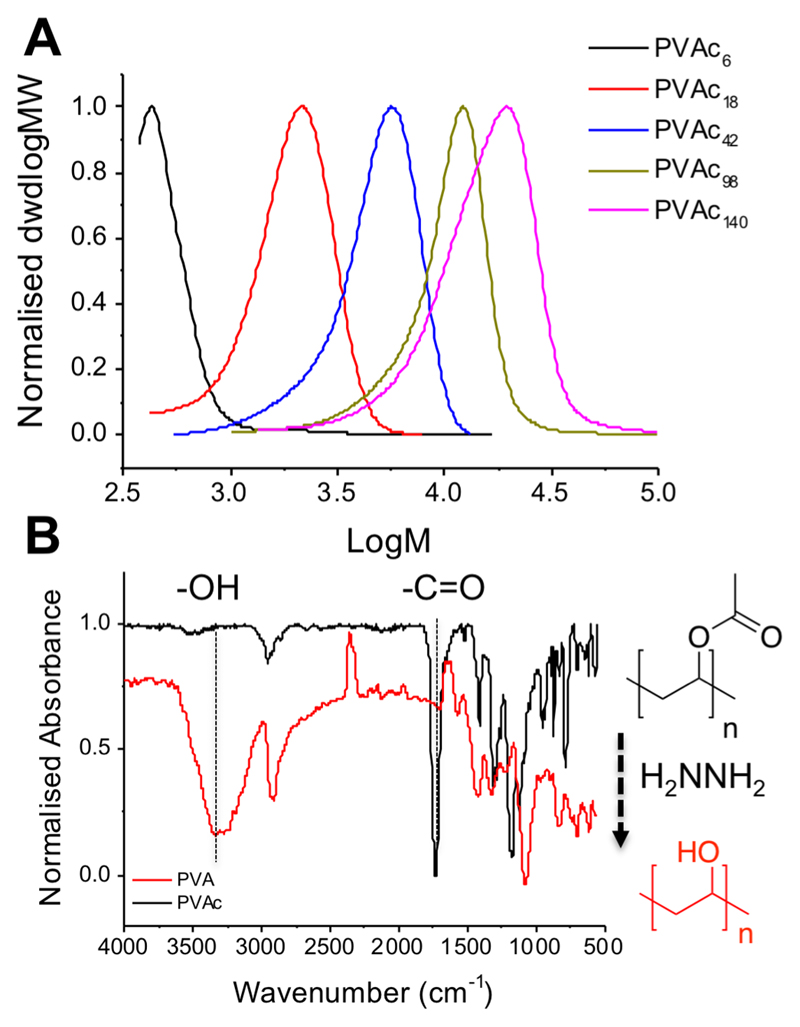
Polymer characterization. (A) SEC analysis of PVAc, dwdlogMW is the differential molecular-weight distribution. (B) Infrared spectra showing removal of C＝O and formation of —OH following hydrazinolysis.

**Scheme 1:**
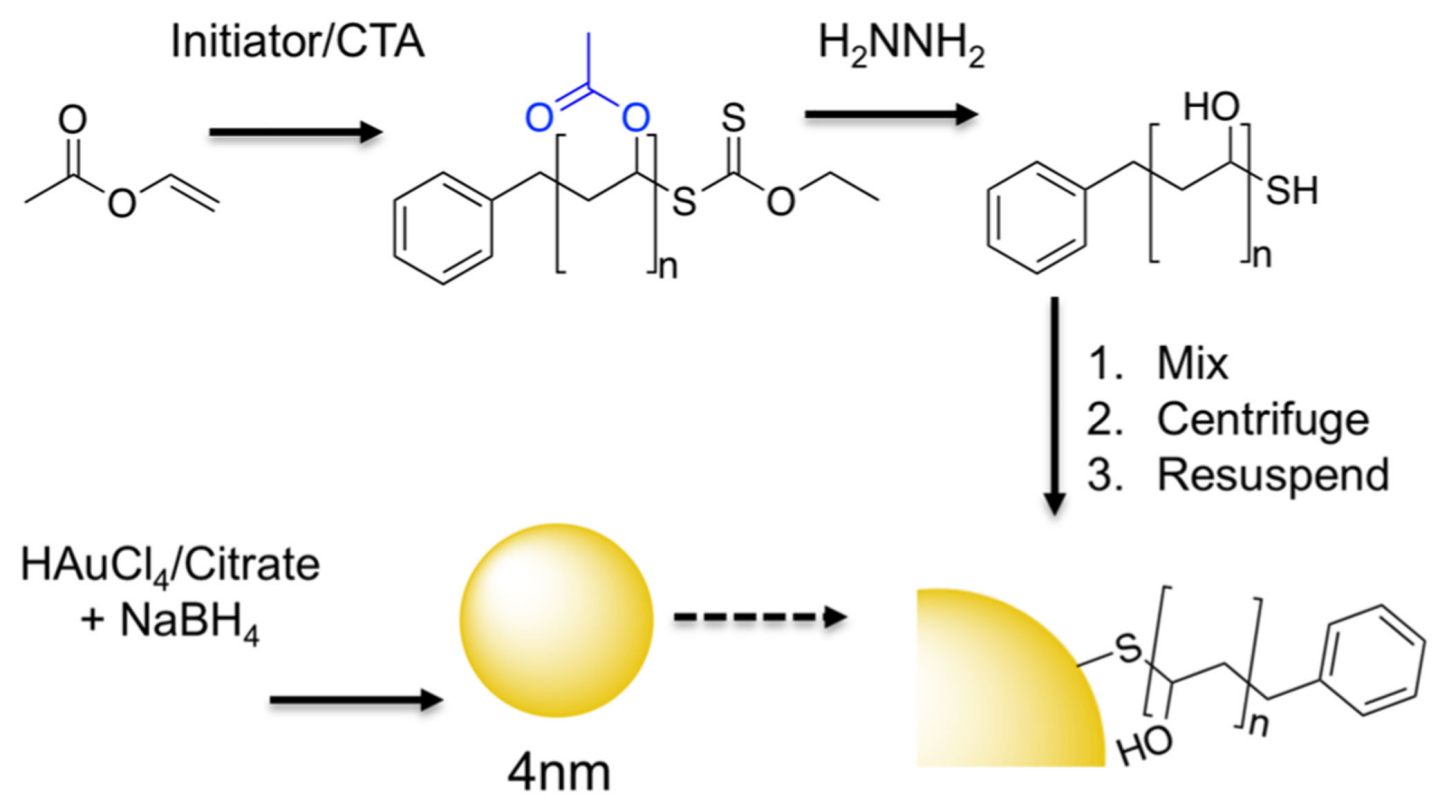
Synthetic Strategy^*a*^ Used for Polymer-Coated Nanoparticles ^*a*^Initiator = 4,4′-azobis(4-cyanovaleric acid); chain-transfer agent (CTA) = *S*-benzyl *O*-ethyl carbondithioate; solvent was dioxane.

To obtain multivalent polymer-coated nanoparticles, RAFT/MADIX polymerization (MADIX, macromolecular design by interchange of xanthates) was exploited to install a protected thiol at *ω*-chain-ends via xanthate chain-transfer agent,^20^,^43^,^45^ essential for a controlled polymerization of vinyl acetate, Scheme 1. Vinyl acetate was polymerized using various ratios of *S*-benzyl *O*-ethyl carbondithioate to give a panel of poly(vinyl acetates)’s (PVAc’s) (see Table 1), which were characterized by ^1^H NMR spectroscopy, SEC (size exclusion chromatography), and IR (infrared) spectroscopy. *M*_n_ values of the resulting polymers were in reasonable agreement with the theoretical values; dispersity values were rather broad as expected for polymers derived from lesser activated monomers^46^ (Figure 1A). These polymers were subsequently hydrolyzed by addition of hydrazine, to remove the acetate protecting groups, which was confirmed by the absence of acetate methyl groups in ^1^H NMR as well as complete removal of carbonyl stretches (1729 cm^−1^) and appearance of hydroxyl (3300 cm^−1^) in the infrared (Figure 1B).

**Figure 2:**
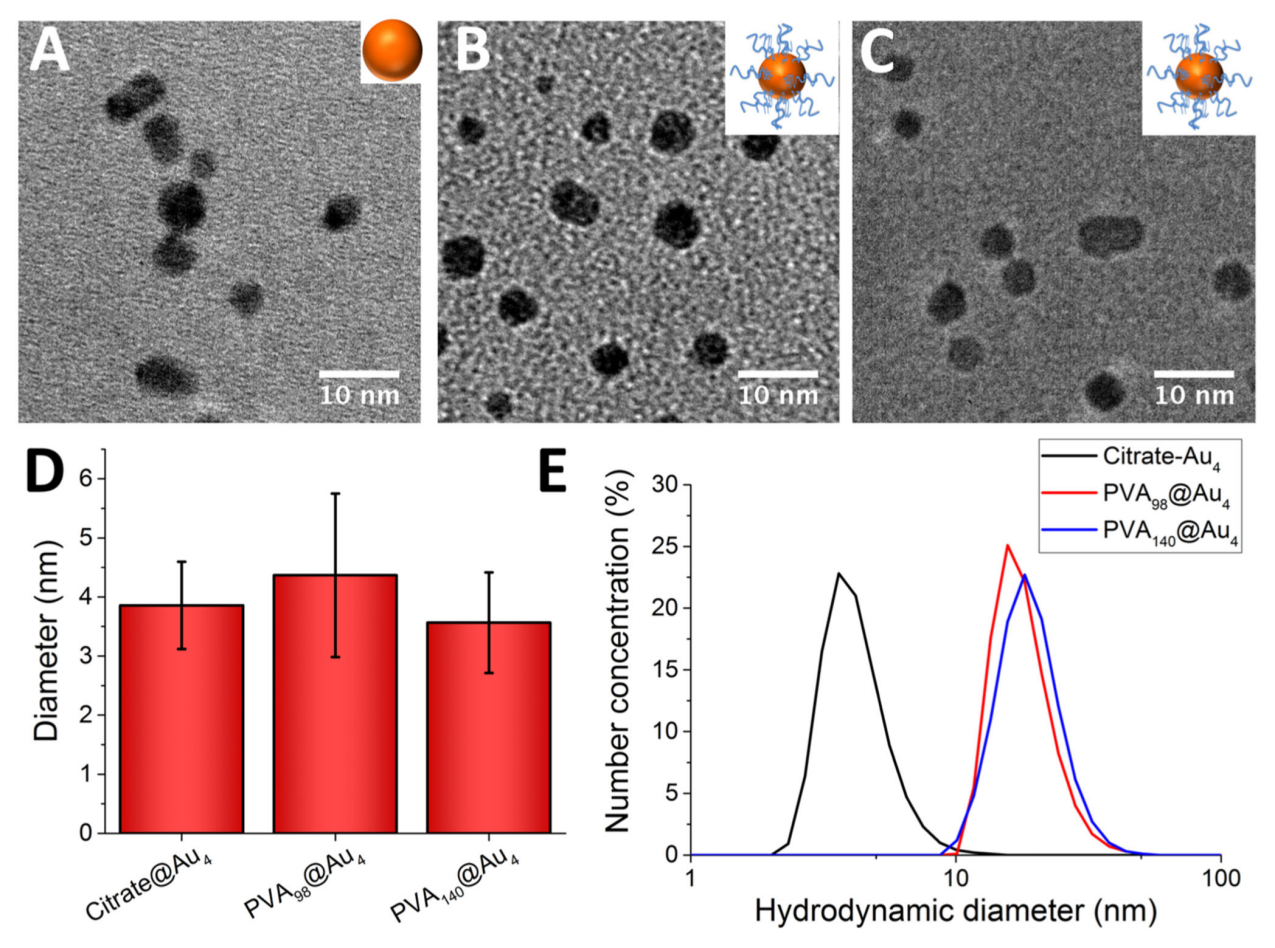
Characterization of nanoparticles. PVA_98_@Au_4_ refers to a DP98 PVA conjugated to a 4 nm gold nanoparticle. (A–C) TEM micrographs of citrate@Au_4_, PVA_98_@Au_4_, and PVA_140_@Au_4_, respectively. (D) Diameter of AuNPs from TEM. Error bars are ±SD from >100 particles. (E) Hydrodynamic diameter of AuNPs (by number particle distribution) from DLS.

Gold nanoparticles (Au_4_; 4 nm diameter) were synthesized by reducing a solution of gold(III) chloride and trisodium citrate with sodium borohydride (see Table 2).^47^ PVAs were immobilized by simple mixing with the citrate-stabilized gold nanoparticle (AuNP) solution, and excess polymer was removed by repeated centrifugation and resuspension cycles. The removal of excess (unbound) PVA was confirmed by IRI assays (see later) on the washings to confirm that there was no residual PVA, and hence free polymer does not interfere with later assays (Supporting Information). The resulting hybrid nanoparticles (PVA@Au_4_) were fully characterized by TEM (transmission electron microscopy, Figure 2A–D), DLS (dynamic light scattering, Figure 2E), XPS (X-ray photo-electron spectroscopy), UV–vis spectroscopy, and TGA (thermogravimetric analysis). From visual analysis (photographs in Supporting Information) it was clear that PVA_6_, PVA_18_, and PVA_42_ did not lead to stable AuNP dispersions, with the coloration of the solutions becoming purple/blue, indicative of surface plasmon resonance (SPR) bands of the gold coupling due to aggregation of the nanoparticles.^48^ These were therefore excluded from further study. For the other polymers, surface functionalization was confirmed by an increase in hydrodynamic diameter from 4 to ~10 nm, and a small red-shift in the SPR_max_. TEM analysis confirmed that the particle size and distribution did not change after polymer functionalization. *ζ* potential measurements showed an increase (from −19.4 to −5 mV) upon polymer coating, due to displacement of the citrate stabilizing ligands, supporting successful surface coating. To enable chemical characterization of the surface, XPS was employed on dried-down nanoparticles (Supporting Information). A significant increase in carbon and sulfur intensities compared to the “naked” citrate particles confirmed the presence of polymer. Finally, TGA confirmed the presence of the polymer coating, with a significant organic mass loss at around 300 °C. Taken together, the above characterization confirmed successful modification of the gold particle surfaces with the poly(vinyl alcohol).

**Figure 3:**
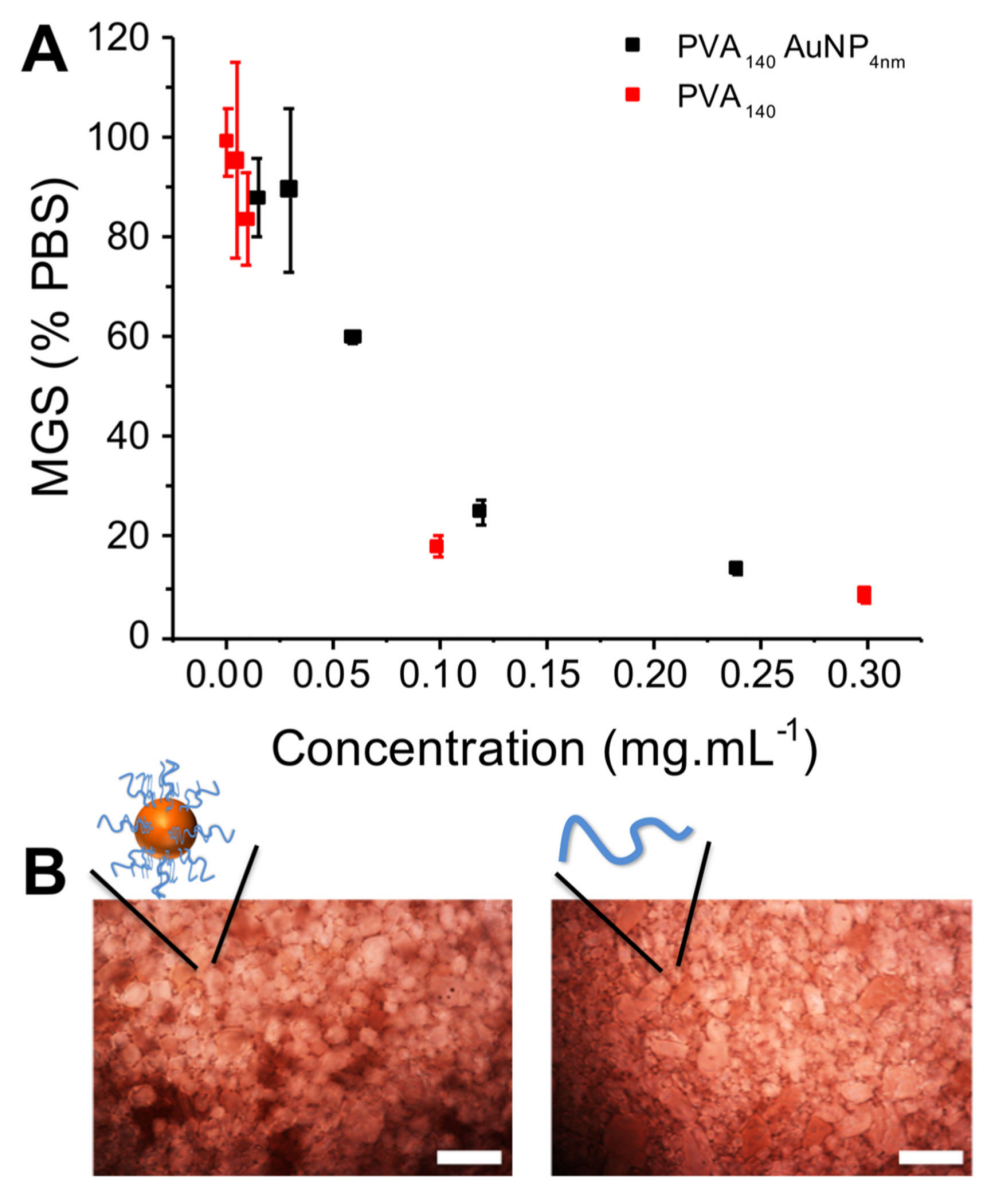
Ice recrystallization inhibition activity analysis. (A) Summary of observed mean largest grain size as a function of concentration. (B) Example cryomicrographs of ice wafers grown with PVA_140_Au_4_ at 0.12 mg mL^−1^ (left), and PVA_140_ at 0.1 mg mL^−1^ (right), scale bars are 100 *μ*m.

These PVA-coated nanoparticles were designed to enable the effect of macromolecular architecture on IRI activity to be probed. Previous results have shown that adding a branch (3-arm versus 2-arm) to polymers of PVA did not lead to any increase in activity.^43^ This is in contrast to a 3-fold increase in molecular weight in linear PVAs which increases activity.^20^ Essentially, previous reports suggest activity is decreased by increased branching at equal total molecular weight, as the additional arm is sterically constrained from aligning with an appropriate crystal face.^32^,^42^ PVA_98_@Au_4_ and PVA_140_@Au_4_ were screened for IRI activity in PBS buffer, based on their observed stability (above). IRI activity was evaluated by monitoring the growth of polynucleated ice crystals (using a “splat” assay^20^,^49^) at subzero temperatures, which enables recrystallization effects to be separated from nucleation (which PVA has an effect on^50^), using an established method.^19^,^20^,^34^ Results are reported as the mean grain size (MGS), relative to a PBS negative control, with smaller grain size values indicating smaller crystals due to more IRI activity, Figure 3.

Linear PVA showed strong IRI activity, inhibiting all growth <1 mg mL^−1^, reinforcing that this is the most potent nonprotein inhibitor known.^9^ Upon testing the gold nano-particles, it was observed that PVA_98_@Au_4_ was not stable under the assay conditions, with some aggregation occurring. This gave rise to potentially false-negative results with it apparently losing activity, and hence these data are excluded (but shown in the Supporting Information for completeness). From the polymers and particles used here only PVA_140_@Au_4_ was stable in the assay, agreeing with previous observations that tuning the surface of polymer-grafted gold particles is chain-length and polymer-type dependent.^48^,^51^,^52^ The IRI activity of the particles is reported in terms of total PVA concentration (calculated from TGA) to enable the activity per chain to be critically compared, Figure 3.

**Figure 4:**
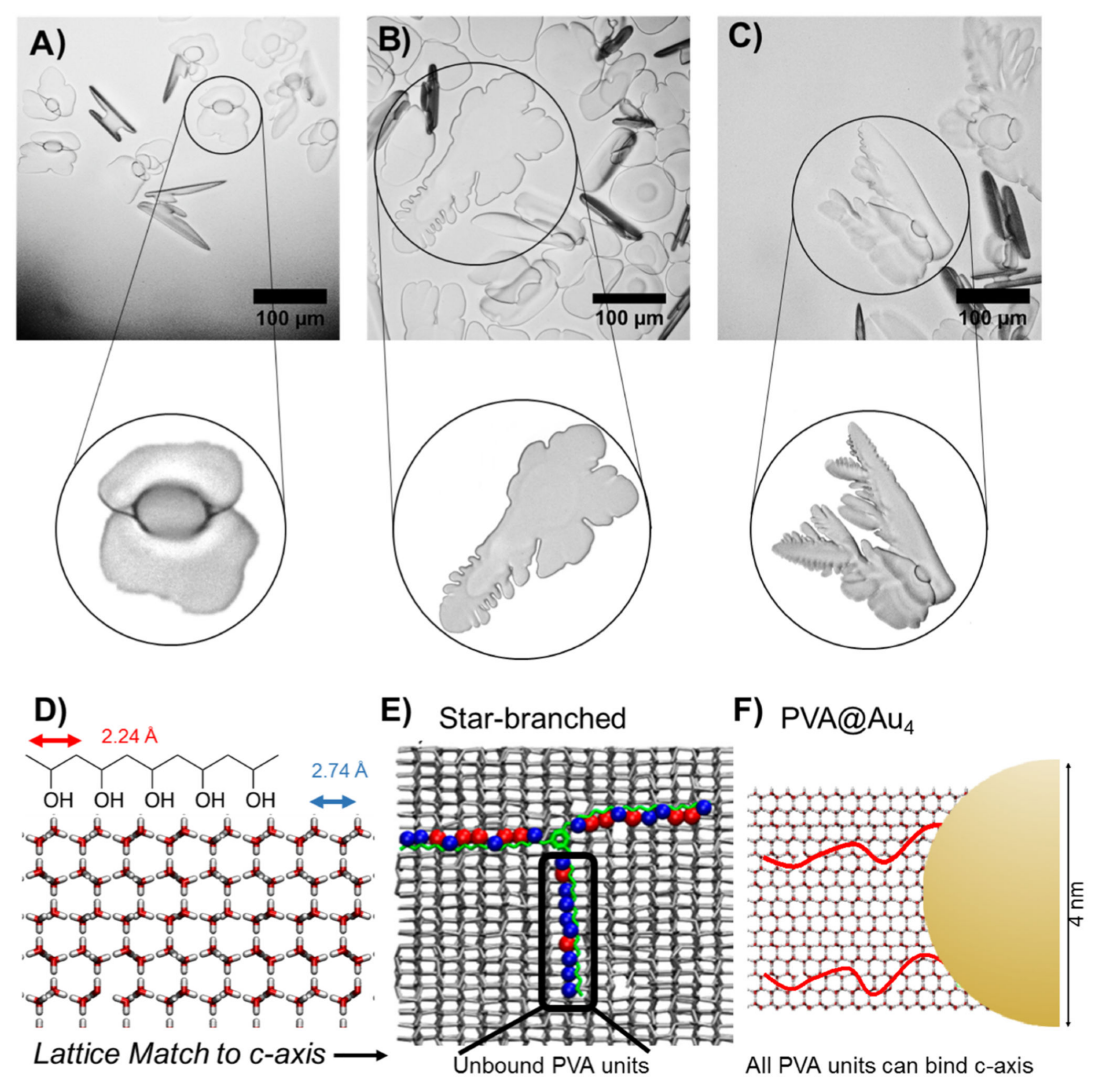
Ice shaping and binding. Cryomicroscopy images in 45% sucrose with zoomed image of crystals of interest. (A) No additive, −6 °C. (B) PVA_140_ 0.32 mg mL^−1^, −5.5 °C. (C) PVA_140_-Au_4_ 0.32 mg mL^−1^, −4 °C. (D) Pattern matching of PVA to the prism plane of ice. (E) (adapted with permission from *J. Phys. Chem. C* **2017**, 121, 26949–26957) shows 3-arm PVA binding to ice, with red circles indicating bound hydroxyls and blue unbound. Highlighted arm has little binding due to being misaligned with lattice. (F) Schematic of gold particle highlighting low density of grafted polymer, does not constrain and hence can find prismatic faces to bind. Scale bars = 100 *μ*m.

Considering the previous reports on varying polymer architecture removing IRI activity, it was remarkable that PVA_140_Au_4_ was observed to retain all IRI, nearly identical (on a per polymer basis) to that of free PVA chains. On a molar basis (assuming one particle is a very large molecule) this would represent a dramatic increase in activity, but such a comparison is not valid for large multivalent systems. The limiting concentration for inhibiting all growth was ~0.1 mg mL^−1^. To explain the retention of activity, how PVA could bind ice must be considered. Molinaro et al. used molecular simulations to hypothesize that PVA can bind to both the primary and secondary prism planes of ice via its hydroxyl groups. The distance between hydroxyls in ice on the primary and secondary prismatic faces is 2.76 and 2.74 Å, respectively, and between consecutive hydroxyls in PVA is 2.92 Å enabling a good fit for pattern matching (Figure 4D).^31^,^32^ However, for the basal faces, the distance is 4.52 Å. For 3-arm (star) PVA, 2 arms can bind prismatic plane, but the third arm is perpendicular to the *c*-axis, cannot form as many hydrogen bonds, and hence has no activity associated (Figure 4E). In the present work, the PVA chains are lightly grafted over a large nanoparticle surface, rather than a specific central conjugation point. From thermogravimetric analysis, relatively low grafting densities are obtained, at 8 chains particle^−1^ (0.1 chains nm^−2^). This low density means the polymers are free to interact with the ice faces, and to target the desirable prism faces unlike in highly grafted systems (such as star polymers^43^ or bottle-brush^42^), Figure 4F.

To demonstrate that the PVA–nanoparticle hybrids were directly interacting with the ice surface, ice shaping experiments were conducted. In this assay, a concentrated sucrose solution (which reduces nucleation and slows growth rates) was used to obtain ice crystals, whose growth could be monitored. For aqueous solutions, rounded disc shaped ice crystals were observed (Figure 4A). However, for both the PVA and PVA_140_@AuNP_4_ faceting was observed and dendritic morphologies observed consistent with ice binding and shaping, as seen for dendronized AFP.^53^

Considering the above data, it is clear that the lightly grafted PVA/AuNP hybrids retain the full activity of the IRI active polymers despite a chain-end being immobilized onto the particle. This confirms that, to assemble larger nanoscopic IRI active materials suitable for application, the overall grafting density and flexibility must be considered to ensure the material can access all ice faces and appears to confirm the simulation/theory evidence for how this apparently simple polymer interacts with the complex ice/water interface.

## Conclusions

Here we have demonstrated the first synthesis and characterization of poly(vinyl alcohol) gold nanoparticle hybrids by using RAFT polymerization and grafting-to methodology. PVA chain lengths were identified which gave colloidally stable particles, due to the relatively low grafting densities achieved by this strategy, and the surface-bound polymers were observed to retain their full ice recrystallization inhibition activity. We hypothesize that the low grafting density on the particle surface is beneficial as it enables the polymers to explore the ice surface so each can engage with prism faces, which have the correct hydroxyl spacings to enable PVA binding. This is in contrast to confined (e.g., 3-armed star) polymers which are sterically constrained so only a small proportion can engage in favorable prism-face interactions. This hypothesis was supported by ice shaping assays with addition faceting/dendritic ice observed for the nanoparticles. These challenge the previous observations, showing that complex architecture can be used to modulate and maintain IRI activity by controlling the PVA density. These results will not only guide the development of more active IRIs, but also enable their application in more fields where their nanoscopic dimensions, or optical properties, are useful, such as in high-resolution imaging.

## Experimental Section

### General

Phosphate-buffered saline (PBS) solutions were prepared using preformulated tablets (Sigma-Aldrich) in 200 mL of Milli-Q water (>18.2 Ω mean resistivity) to give [NaCl] = 0.138 M, [KCl] = 0.0027 M, and pH 7.4. Vinyl acetate (>99%), 4,4′azobis(4-cynaovaleric acid) (>98%), and benzyl bromide (98%) were purchased from Sigma-Aldrich. Vinyl acetate was filtered through a plug of basic alumina to remove inhibitors prior to use. The 4,4′azobis(4-cynaovaleric acid) was recrystallized from methanol and stored at −18 °C in the dark. Potassium ethyl xanthate (98%) was purchased from Alfa Aesar. *S*-benzyl *O*-ethyl carbondithioate was synthesized as previously described.^20^

### Physical and Analytical Methods

^1^H and ^13^C NMR spectra were recorded on Bruker Avance III HD 300 MHz, HD 400 MHz, or HD 500 MHz spectrometers using deuterated solvents obtained from Sigma-Aldrich. Chemical shifts are reported relative to residual nondeuterated solvent. All size exclusion chromatography (SEC) data were recorded in THF on Agilent 390-LC MDS instruments equipped with differential refractive index (DRI) detectors. Systems were equipped with 2× PLgel Mixed D columns (300 × 7.5 mm) and a PLgel 5 *μ*m guard column. The eluents are THF with 2% TEA (triethylamine) and 0.01% BHT (butylated hydroxytoluene). All samples were run at 1 mL min^−1^ at 50°C. Poly(methyl methacrylate) standards (Agilent EasyVials) were used for calibration. Analyte samples were filtered through a nylon membrane with 0.22 *μ*m pore size before injection. Respectively, experimental molar mass (*M*_n,SEC_) and dispersity (*Đ*) values of synthesized polymers were determined by conventional calibration using Agilent GPC/SEC software.

Nanoparticle size and dispersity was measured by transmission electron microscopy (TEM), UV–vis spectroscopy, and size measurements by dynamic light scattering (DLS). DLS was performed on a Malvern Instruments Zetasizer Nano-ZS instrument with 4 mW HeNe laser 632.8 nm. UV–vis spectroscopy was performed on a BioTek Synergy HT microplate reader. TEM was performed on a JEOL 2100 LaB6 high-resolution microscope. X-ray photoelectron spectroscopy (XPS) was carried out on the Kratos Axis Ultra instrument with a delay-line detector. TGA was performed on a Metter Toledo STAR^e^ DSC/TGA instrument under nitrogen from 30 to 600 °C using standard alumina 70 *μ*L crucibles and a blank in each run.

Sucrose assays were performed on, and ice wafers were annealed on, a Linkam biological cryostage BCS196 instrument with T95-Linkpad system controller equipped with a LNP95-liquid nitrogen cooling pump, using liquid nitrogen as the coolant (Linkam Scientific Instruments UK, Surrey, UK). An Olympus CX41 microscope equipped with a UIS-2 20*x*/0.45/∞/0-2/FN22 lens (Olympus Ltd., Southend on sea, UK) and a Canon EOS 500D SLR digital camera was used to obtain all images. Image processing was conducted using ImageJ software, which is freely available from http://imagej.nih.gov/ij/.

### Ice Recrystallization Inhibition Assay

A 10 mL droplet of polymer in PBS solution is dropped from 1.4 m onto a glass microscope coverslip, which is on top of an aluminum plate cooled to −78 °C using dry ice. The droplet freezes instantly upon impact with the plate, spreading out and forming a thin wafer of ice. This wafer is then placed on a liquid nitrogen cooled cryostage held at −8 °C. The wafer is then left to anneal for 30 min at −8 °C. Three photographs are then taken of the wafer in different locations at 20× zoom under cross polarizers. The number of crystals in the image is counted, again using ImageJ software, and the area of the field of view divided by this number of crystals to give the average crystal size per wafer, reported as a percent of area compared to PBS control.

### Sucrose Sandwich Ice Shaping Assay

Samples dissolved in PBS buffer containing 45% sucrose were sandwiched between two glass coverslips and sealed with immersion oil. Samples were cooled to −50 °C. The temperature was then increased to −8 °C and held for 1 h to anneal. The samples were then heated at 0.05 °C min^−1^ until few ice crystals remained and then cooled at 0.05 °C min^−1^, and the shape of ice crystals was observed. Micrographs were obtained every 0.1 °C.

## Synthetic Section

### Synthesis of 4 nm Gold Nanoparticles and Subsequent Surface Conjugation of Polymer

The 4 nm citrate-stabilized AuNPs were synthesized as described by Ieong et al.^47^ A 240 mL portion of a 0.21 mmol L^−1^ (0.08 mg mL^−1^), aqueous solution of HAuCl_4_ was prepared at room temperature in glassware washed with aqua regia [CAUTION. HANDLE WITH CARE]. A 13.8 mg (0.05 mmol) portion of trisodium citrate was added, followed by 5 mL of an ice-cold 0.1 M (0.5 mmol, 18.5 mg) solution of NaBH_4_. The solution was stirred at room temperature overnight. Small AuNPs were isolated by taking the supernatant after centrifugation at 13.2k RPM for 10 min at 25 °C. A 1 mg portion of polymer was added to 1 mL of Au_4_, and the solution was agitated for 60 min at room temperature. These solutions were washed three times and concentrated in Amicon Ultra 0.5 centrifugal filter units with an Ultracel-30 membrane before being redispersed in the same volume of PBS.

## Supplementary Material

The Supporting Information is available free of charge on the ACS Publications website at DOI: 10.1021/acs.langmuir.8b01952.

Additional synthesis details and figures including ^1^H NMR spectra, FTIR spectra, UV–vis spectra, photographs, TGA analysis, XPS survey scans, and IRI activity assessment (PDF)

Supp Info

## Figures and Tables

**Table 1:** Poly(vinyl acetate) Synthesized in This Study

polymer^a^	[M]/[CTA]^b^ (−)	conversion^c^ (%)	*M*_n_ (theoretical)^d^ (g mol^−1^)	*M*_n_ (SEC)^e^ (g mol^−1^)	*Ð*^e^ (−)
PVAc_6_	10	72	600	500	1.41
PVAc_18_	40	59	2000	1600	1.24
PVAc_42_	60	68	3500	3600	1.38
PVAc_98_	100	45	3900	8500	1.37
PVAc_140_	250	56	12 000	12 000	1.53

a Sample names are determined according to the number-average degree of polymerization (DP) determined by SEC.

b Monomer to RAFT agent molar ratio.

c Determined by ^1^H NMR using mesitylene as an internal standard.

d Determined from feed ratio and conversion.

e Determined by SEC.

**Table 2:** Gold Nanoparticles Synthesized in This Study

particle	*ζ* potential^a^ (mV)	SPR_max_^b^ (nm)	diameter_TEM_^c^ (nm)	diameter_DLS_^d^ (nm)
citrate@Au_4_	−19.4 ± 2.7^e^	510	3.9 ± 0.74	4.37
PVA_98_-Au_4_	−5.54 ± 0.6^f^	522	4.4 ± 1.4	18.2
PVA_140_-Au_4_	−4.49 ± 0.4^f^	521	3.6 ± 0.85	19.4

a Averaged over 3 measurements.

b Maximum of SPR peak determined by UV–vis spectroscopy.

c Taken as the average of 100 particles by TEM.

d Determined by dynamic light scattering, distribution of size by number of particles.

e Solution pH measured as 8.9.

f AuNPs suspended in PBS, pH 7.25.
