# Supplementary material for: Multivalent Presentation of Ice Recrystallization Inhibiting Polymers on Nanoparticles Retains Activity
Source: Langmuir. Author manuscript; Available in PMC 2020 Feb 22. (PMC6354916; doi:10.1021/acs.langmuir.8b01952)
Supplement: Supp Info [file NIHMS79875-supplement-Supp_Info.pdf]

Electronic Supporting Information for;

Multivalent Presentation of Ice Recrystallization Inhibiting Polymers on Nanoparticles Retains Activity

Christopher Stubbs,[a]‡ Laura E. Wilkins,[a]‡ Alice E. R. Fayter,[a] Marc Walker,[b] and Matthew I. Gibson[a,c]*

[a] Department of Chemistry, University of Warwick, UK CV4 7AL

[b] Department of Physics, University of Warwick, UK CV4 7AL

[c] Warwick Medical School, University of Warwick, UK CV4 7AL

* Corresponding author. Email m.i.gibson@warwick.ac.uk

‡ These authors contributed equally

Synthesis of *S*-benzyl *O*-ethyl carbondithioate.

Acetone (40 mL) was added to a round bottom flask equipped with a stirrer bar. Potassium ethyl xanthogenate (2 g, 0.0124 mol, 1 eq.) was added and left to dissolve for 30 minutes. Benzyl bromide (2.13 g, 0.0124 mol, 1eq.) was added and stirred for 24 hours at 50 °C. The solution was filtered and the filtrate concentrated *in vacuo*. The resulting yellow oil was purified on a column of silica with DCM as eluent. Yield 1.26 g 48 %. ^{1}H NMR (300 MHz, $CDCl_3$): **δ** = 1.3 ($CH_3CH_2OC(S)S$, t, 3H), 4.3 ($OC(S)SCH_2$, s, 2H), 4.6 ($CH_3CH_2OC(S)S$, q, 2H), 7.2 ($C(S)SCH_2C_6H_5$, br, 5H). ^{13}C NMR (400 MHz, $CDCl_3$): **δ** = 14 (CH_3CH_2), 40 ($C(S)SCH_2$), 70 (CH_3CH_2), 127 (*para* CH), 128 (*meta* CH), 129 (*ortho* CH), 213 ($OC(S)S$).

Synthesis of poly(vinyl acetate)$_{98}$.

As a representative example, vinyl acetate (3 g, 3.21 mL, 34 mmol, 1000 eq), *S*-benzyl *O*-ethyl carbondithioate (0.074 g, 0.34 mmol, 10 eq.), 4,4'azobis(4-cyanovaleric acid) (0.0098 g, 0.034 mmol, 1 eq.) and mesitylene (0.1 g, 0.086 mL) were added to a 10ml vial and sealed with a subaseal. The solution was left to degas under nitrogen for 15 minutes, before being placed into an oil bath at 68 °C. The reaction was left for 24 hours before being plunged into liquid nitrogen. Poly(vinyl acetate) was recovered as a sticky yellow oil after precipitation into diethyl ether.

Representative characterization data for poly(vinyl acetate)$_{98}$:

^{1}H NMR (400 MHz, $CDCl_3$) δ = 4.90 – 4.70 ($CH_2CH(OOCH_3)$, br, 1H), 2.07 ($CH_2CH(OOCH_3)$, br, 3H), 1.90-1.60 ($CH_2CH(OOCH_3)$, br, 2H).

IR C-H 2954 cm^{-1}, C=O 1729 cm^{-1}, C-O 1407 cm^{-1}.

M_n^{SEC}(THF) = 8500 Da, M_w/M_n = 1.37

Synthesis of poly(vinyl alcohol)$_{98}$.

As a representative example, poly(vinyl acetate) (1 g) was dissolved in methanol (5 mL) in a round bottom flask and stirred until dissolved. Hydrazine hydrate solution (15 mL, 78-82 % in water) was added and the reaction heated to 50 °C in an oil bath for 24 hours. The solution was cooled and diluted with water (50ml) before being purified by dialysis (100-500 MWCO) followed by lyophilization to form a white powder. Representative characterization data for poly(vinyl alcohol)$_{98}$:

^{1}H NMR (400 MHz, D_2O) δ = 4.00 – 3.80 (CH_2C*H*(OH), br, 1H), 1.80 – 1.30 (C*H*$_2$CH(OH), br, 2H).

IR O-H 3000-3400 cm^{-1}, C-H 2917 cm^{-1}, C-O 1411 cm^{-1}.

Modified Sucrose Sandwich Ice Shaping Assay

Samples dissolved in PBS buffer containing 45% sucrose were sandwiched between two glass coverslips and sealed with immersion oil. Samples were cooled to −50 °C on a Linkam Biological Cryostage BCS196 with T95-Linkpad system controller equipped with a LNP95-Liquid nitrogen cooling pump, using liquid nitrogen as the coolant (Linkam Scientific Instruments UK, Surrey, U.K.). The temperature was then increased to −8 °C and held for 1 hour to anneal. The samples were then heated at 0.5 °C.min^{-1} until few ice crystals remained and then cooled at 0.05 °C.min^{-1} and the shape of ice crystals observed. Micrographs were obtained every 0.1 °C using an Olympus CX41 microscope equipped with a UIS-2 20x/0.45/∞/0−2/FN22 lens (Olympus Ltd., Southend on sea, U.K.) and a Canon EOS 500D SLR digital. Image processing was conducted using ImageJ.

Ice Shaping Image Enhancement

Ice crystal images contrast was modified in ImageJ to show faceting more clearly, followed by removal of the background using photoshop mix. This enabled the zoomed in images in Figure 4 to be shown. These are directly taken from the images shown in the main panel in Figure 4.

Additional Polymer Characterization

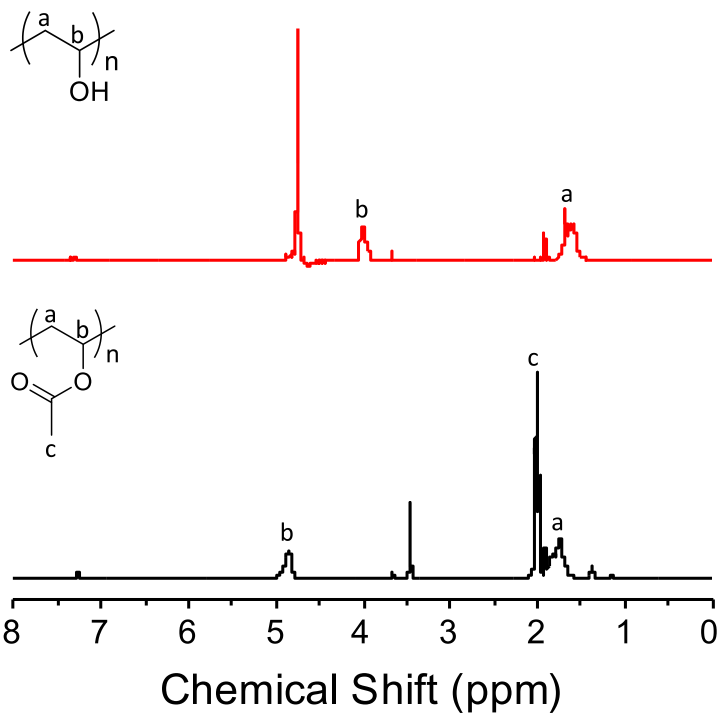

Figure S1. ^{1}H NMR spectra showing removal of acetate groups (peak c) from PVAc's

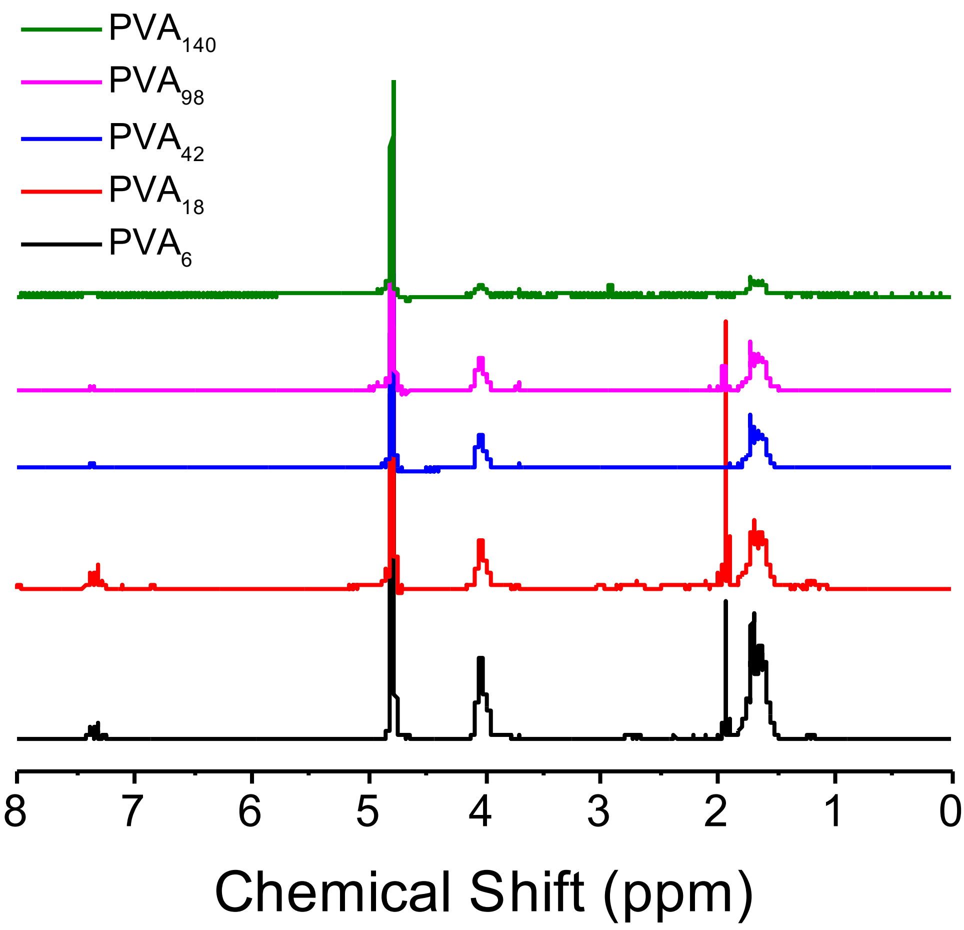

Figure S2: ^{1}H NMR spectra of the PVA's synthesized in this study.

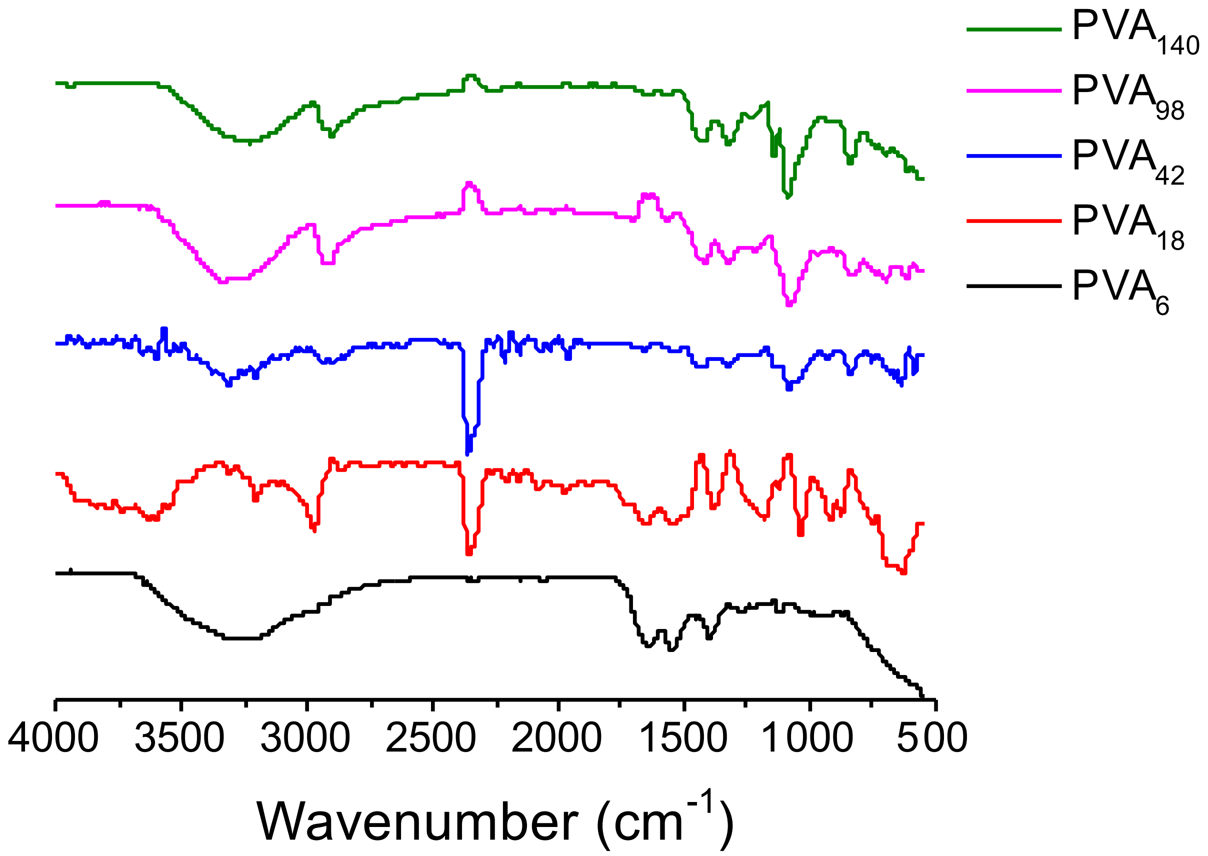

Figure S3: FTIR of the PVAs synthesized in this study (post-hydrolysis of the acetate group)

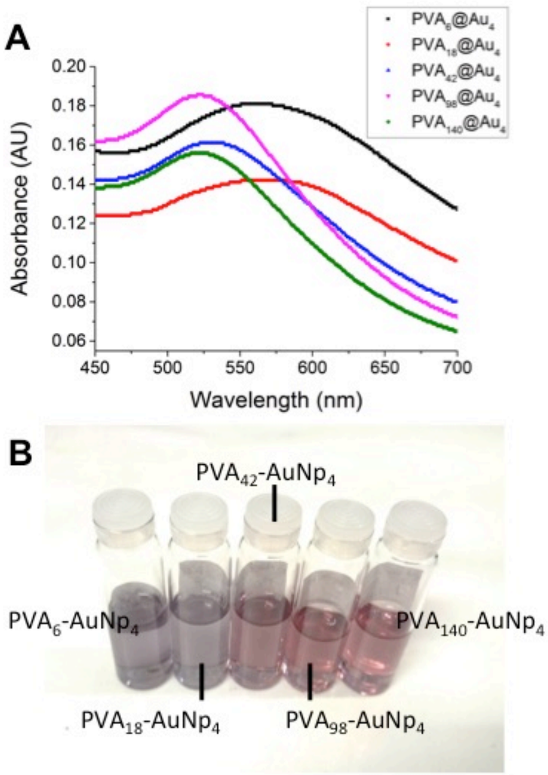

Figure S4. Gold nanoparticle characterization in water. A) UV-visible spectra; B) photographs showing colouration of polymer coated particles.

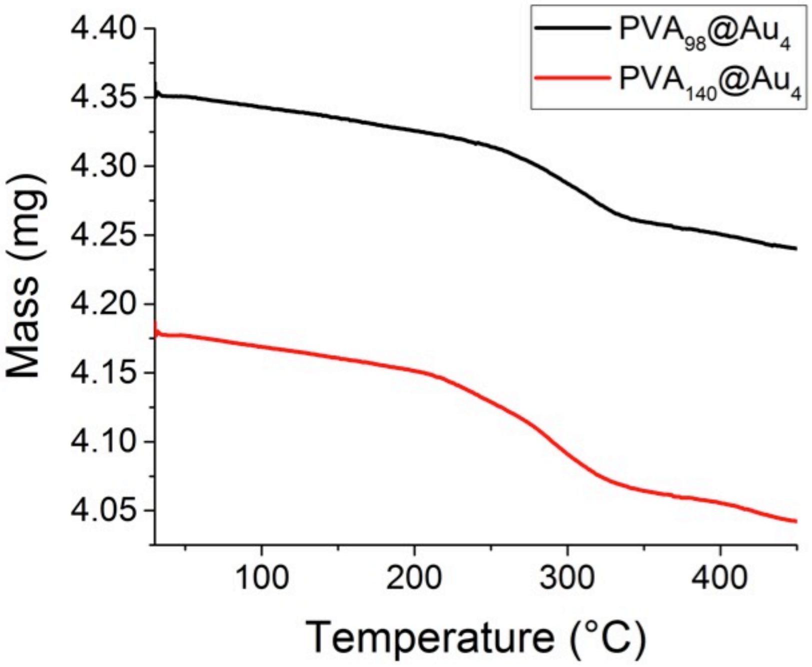

Figure S5. TGA analysis of polymer-coated gold nanoparticles

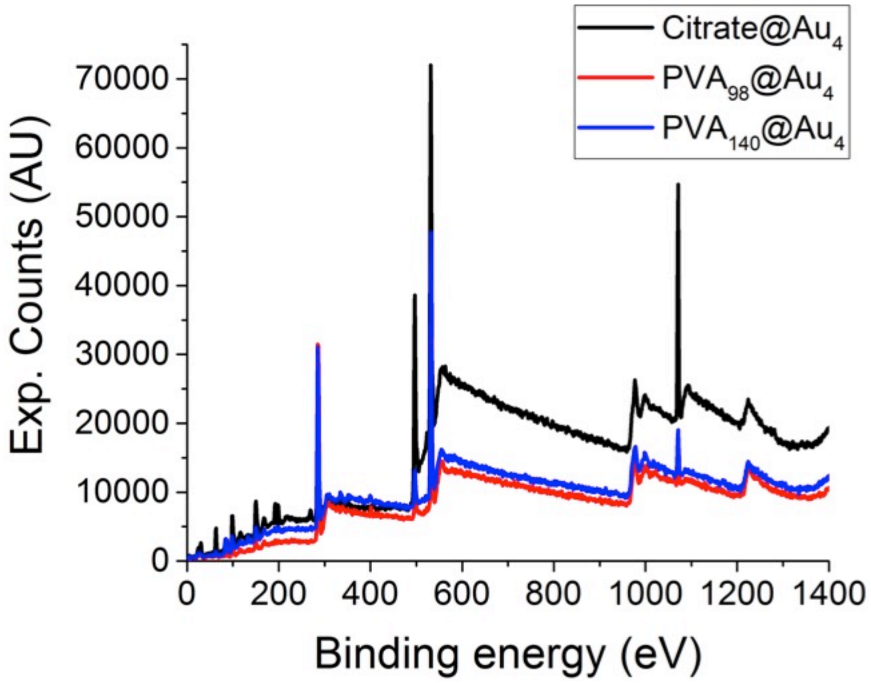

Figure S6. XPS survey scans of nanoparticles.

Additional IRI Data

As discussed in the main text, PVA98@Au4 was not colloidally stable in the IRI assays. This aggregation reduced the observed activity as a false-negative results. The 'splat' assay data on this is included here for completeness.

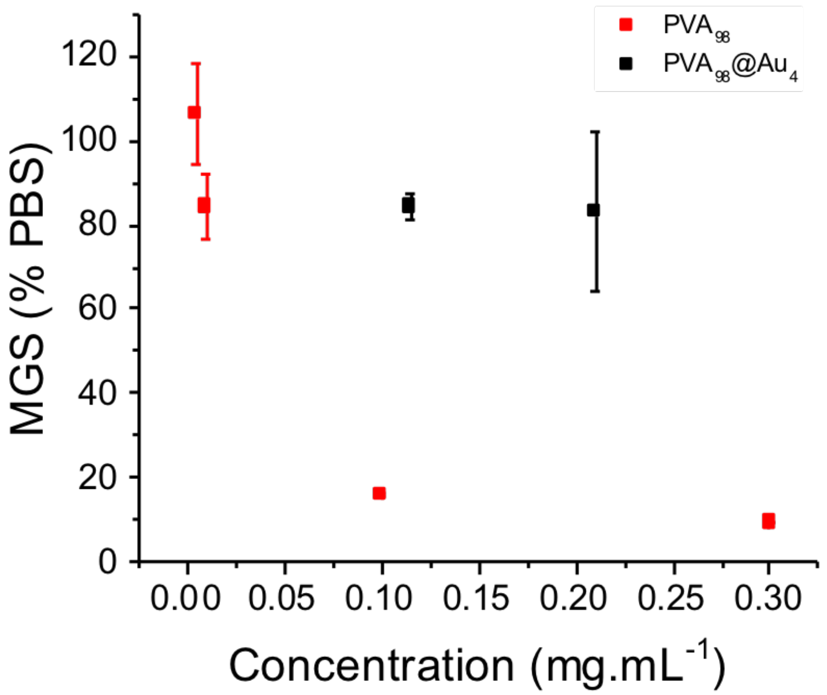

Figure S7. IRI activity assessment of $PVA_{98}@Au_4$, which was not colloidally stable.

To ensure there is no residual polymer, the washing from the PVA@AuNP purification cycles were analyzed by the Splat assay, Figure S8. PVA retains IRI activity down to ~ 0.1 mg.mL^{-1}. PVA was added at 1 mg.mL-1 concentration during the synthesis. Figure S8 shows that 3 washing are required to remove PVA such that any (small) residual amounts do not interfere with the IRI activity measurements of the AuNPs.

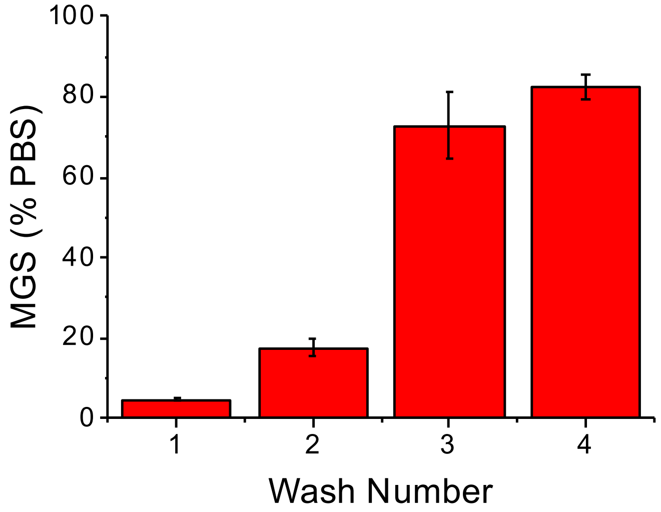

Figure S8. Residual IRI activity (determined *via* splat test) of the washings following centrifugal purification of the PVA@AuNPs. Larger numbers indicate less activity. Errors bars are the standard deviation from 3 sets of measurements.
